# The grass isn’t always greener: The effects of cannabis on embryological development

**DOI:** 10.1186/s40360-016-0085-6

**Published:** 2016-09-29

**Authors:** Joseph Friedrich, Dara Khatib, Keon Parsa, Ariana Santopietro, G. Ian Gallicano

**Affiliations:** Department of Biochemistry and Molecular & Cellular biology, Georgetown University Medical Center, 3900 reservoir Rd. NW, Med/Dent Building NE205, Washington DC, 20057 USA

**Keywords:** Cannabis, Pregnancy, Folic acid, Pre-implantation, Metabolism, Neurogenesis, Angiogenesis

## Abstract

With the increasing publicity of marijuana due to recent legislation, it is pertinent that the effects of fetal exposure to the drug are assessed. While *in utero* cannabis exposure has been associated with early pregnancy failure, birth defects and developmental delay, the mechanisms of such outcomes are largely unexplained. Furthermore, the use of cannabinoids in cancer treatment via growth inhibition and apoptosis may indicate how cannabis exposure likely harms a growing fetus. Cannabinoid signaling is required for proper pre-implantation development, embryo transport to the uterus, and uterine receptivity during implantation. In post-implantation development, cannabinoid signaling functions in a multitude of pathways, including, but not limited to, folic acid, VEGF, PCNA, MAPK/ERK, and BDNF. Disrupting the normal activity of these pathways can significantly alter many vital *in utero* processes, including angiogenesis, cellular replication, tissue differentiation, and neural cognitive development. This paper aims to demonstrate the effects of cannabis exposure on a developing embryo in order to provide a molecular explanation for the adverse outcomes associated with cannabis use during pregnancy.

## Background

It has long been established that smoking tobacco during pregnancy causes increased risk of miscarriage, increased placental problems, reduction of birth weight, and a variety of birth defects [1]. In light of the recent legalization of marijuana in Colorado, Washington, Alaska and Washington, D.C., we felt it important to establish and publicize the causative relationship between cannabis usage and embryological outcomes. The main psychoactive cannabinoid in marijuana is delta-9-tetrahydrocannabinol (THC), which has a half-life of approximately 8 days in fat deposits and can be detected in blood for up to 30 days before becoming entirely eliminated from the blood [2]. These characteristics act as a direct risk factor to the developing embryo, as the maternal tissues act as reservoirs for THC and other cannabinoids.

Certain drugs cross the placenta to reach the embryo in the same manner as oxygen and other nutrients [3]. Drugs consumed during pregnancy can act directly on the embryo, or they can alter placental function, which is critical for normal growth and development. Ingestion of drugs can interfere with these functions, resulting in compromised fetal development and growth [3]. THC readily crosses the placenta, which, in conjunction with slow fetal clearance, results in prolonged fetal exposure to THC, even after consumption is discontinued [2].

**Fig. 1:**
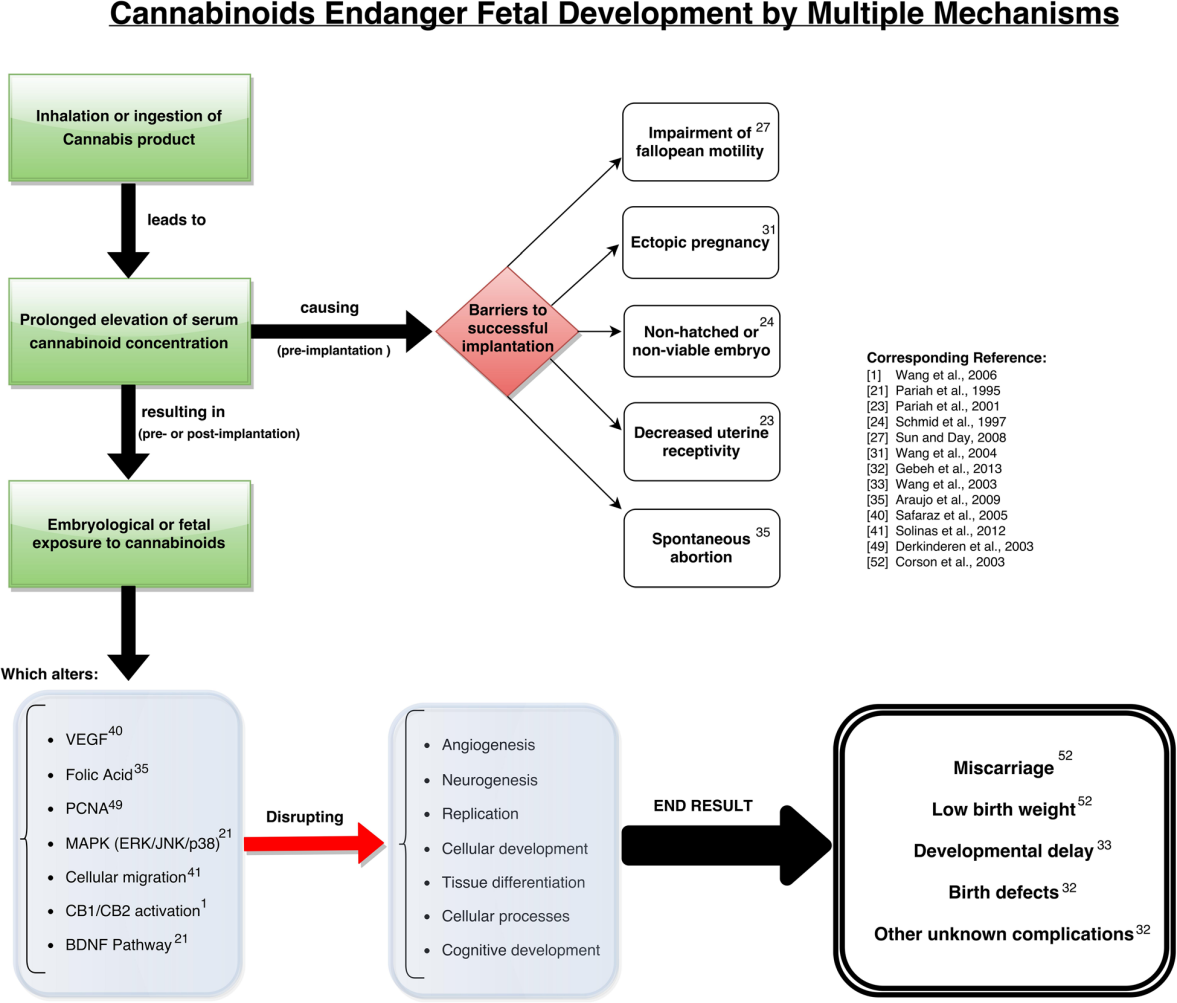
Overview of cannabinoid action on fetal developmental mechanisms. A flowchart of the proposed mechanisms by which cannabis affects embryological and fetal development. Note that many “end result” outcomes were observed in animal models

The use of marijuana in early pregnancy is associated with many of the same risks as tobacco, including miscarriage, congenital malformations, and learning disabilities [4]. Adverse effects of marijuana use during pregnancy have been exacerbated over the years, as THC levels in marijuana have increased nearly 25-fold since 1970 [5]. This paper looks to examine recent studies on cannabinoids and embryonic development in order to establish the mechanisms through which these cannabinoids act. A full overview of these mechanisms and downstream effects are provided in Fig. 1 and Table 1.

**Table 1:** A summary table of cannabinoid effects on various cellular processes

Specific Protein or Process	Effect of Cannabinoid on Protein or Process	Downstream Effect	Study Method	Study
Angiogenic proteins	Administration of the cannabinoid JWH altered the expression of 10 genes which are all directly or indirectly related to the VEGF pathway	Disruption of normal angiogenesis	In vivo*.* Mice bearing s.c. gliomas injected with JWH	Blazquez et al., 2004. [36]
Apoptosis (Epithelial tumor)	Treatment of PDV.C57 cells with the cannabinoid WIN induces apoptosis	Premature cellular death	In vitro*.* Treatment of skin tumor cells with WIN	Casanova et al., 2003. [43]
Apoptosis (HUVEC)	Cannabinoid-induced HUVEC death occurred by apoptosis	Premature cellular death	In vitro*.* HUVEC cells treated with WIN	Blazquez et al., 2003. [42]
Cellular Migration	Cannabidiol treatments causes a decrease in cell migration in a concentration dependent manner.	Reduced cellular migration, possible impairment of cellular differentiation	In vivo and In vitro*.* Cannabidiol treatment of HUVEC cells and treatment of C57/BL6 mice	Solinas et al., 2012. [41]
Ceremides	The ceramide-dependent cannabinoid-induced inhibition of VEGFR-2 activation is found in cultured glioma cells	Disruption of cellular messaging	In vitro*.* Mice bearing s.c. gliomas injected with JWH and WIN	Blazquez et al., 2004. [36]
Folic acid	Chronic Cannabidiol consumption results in a decrease in folic acid uptake, while acute Cannabidiol consumption results in no effect on folic acid uptake	Disruption of DNA replication, possible neural tube defects	In vitro*.* Cannabidiol treatment of BeWo cells incubated with folic acid	Araújo et al., 2009. [35]
PCNA	Treatment of LNCaP cells with the cannabinoid WIN results in a significant decrease in protein expression of PCNA in a dose dependent manner.	Disruption of DNA replication	In vitro*. LNCaP* cancer cells treated with WIN	Sarfaraz et al., 2005. [40]
VEGF	Cannabidiol significantly inhibits the VEGF induced outgrowth of capillary-like structures	Disruption of normal angiogenesis	In vitro*.* Inhibition of the in-vitro angiogenesis in HUVEC Cells	Solinas et al., 2012. [41]

*Abbreviations: HUVEC* Human umbilical vein endothelial cell, *JWH* JWH-133 (Cannabinoid and CB2 agonist), *LNCaP* androgen-sensitive human prostate adenocarcinoma cells, *PCNA* Proliferating cell nuclear antigen, *S.c.* Subcutaneous, *VEGF* Vascular endothelial growth factor, *WIN* WIN-55,212-2 (Cannabinoid and CB1 agonist)

### Methods

To compile the literature analyzed in this review, MEDLINE was searched for English language articles between 1975 and 2015. First, exploratory searches were conducted using the key words *cannabinoid* AND *embryo* AND *development*. Searches were then conducted using combinations of the Medical Subject Heading (MeSH) terms *Cannabinoids*, *Cannabis*, *Marijuana*, and *Fetal Development*. We reviewed the identified articles for applicability and selected the articles that included the in vitro or in vivo effects of altered cannabinoid signaling, in vivo concentrations of cannabis following cannabinoid use or infusion, and epidemiological effects of cannabis use. Additional articles were selected from the bibliographies of searched articles.

### Metabolism, physiologic concentration, and placental transfer

The pharmacokinetics of cannabis has been shown to vary depending on the route of administration, which can impact the rate of drug absorption [6]. Smoking is a preferred method of consumption for many cannabis users because of its rapid drug delivery and its rapid manifestation in physiological effects [7]. When smoking, drug exposure varies with the number and duration of puffs, as well as the hold time, inhalation volume, or smoking topography [8–10]. Studies have been conducted with various dosages of marijuana cigarettes to determine the concentrations of THC and its metabolites both immediately after smoking and several days afterwards.

In a study by Huestis et al., a computer-paced smoking procedure was implemented to control the number of puffs, length of inhalation, and time between puffs in several human subjects. A low dosage marijuana cigarette was defined as having 16 mg THC (1.75 %), while a high-dosage cigarette was defined as having 34 mg THC (3.55 %) [11]. Immediately after inhalation of the marijuana cigarette, THC concentrations in blood were measured via a continuous blood-withdrawal pump, which collected blood at a rate of 5 ml/min. Mean THC concentrations were 7.0 ± 8.1 ng/mL and 18.1 ± 12.0 ng/mL upon single inhalation of low and high dose marijuana cigarettes, respectively [11]. The THC concentrations increased rapidly and reached peaks of 84.4 ng/mL and 162.2 ng/mL for low and high dosage marijuana cigarettes, respectively, at 9 min in both cases [12]. A similar study found that smoking two marijuana cigarettes with approximately 25 mg THC per cigarette (2.57 %) elevated plasma concentrations of THC by approximately 300 ng/mL [7].

Huestis et al define a high dosage of THC as 34 mg, however, this underestimates the potency cannabis that is sold and used today. In 2010, researchers working with the National Institute on Drug Abuse analyzed 46,211 samples of cannabis confiscated by domestic law enforcement between 1993 to 2008 [13]. The data reveal a steady increase in average concentrations of THC, increasing from 3.4 % (34 mg per cigarette) in 1994 to 8.8 % (84 mg per cigarette) in 2008 [13]. The mean concentration of THC in hashish, a popular preparation of cannabis, was found to range to be from 12.69 to 15.56 % during the same period [13].

Ingestion of cannabinoids by the oral route is another common method, especially for therapeutic applications. Ingestion of cannabinoids results in slower absorption with lower, more-delayed peak THC concentrations [6, 14]. A study by Ohlsson et al measured THC concentrations based on GC/MS experiments, and found that the peak THC concentrations ranged from 4.4 to 11 ng/mL, which occurred 1–5 h following ingestion of 20 mg of THC in a chocolate cookie [6]. The lower peak of oral ingestion of THC versus smoking can be attributed to several factors, including variable absorption, breakdown of the drug in the stomach, as well as first–pass metabolism to active 11–OH-THC and inactive metabolites in the liver [11].

However, Ohlsson et al underestimated the amount of THC in products available to consumers, as the advent of legalization has given rise to a niche market of cannabis products with artificially elevated concentrations of cannabinoids [15, 16]. Today, legally available edible preparations of cannabis, for example, baked goods or chocolate bars sold in San Francisco, California, are found to contain THC content ranging from 1 mg of THC to greater than 1200 mg of THC per item when analyzed via high-performance liquid chromatography [17].

Once cannabinoids are present in the bloodstream, their lipophilic nature allows them to readily cross the placenta and enter the fetal bloodstream [18, 19]. Studies conducted on pregnant dogs demonstrate that THC is deposited in fetal fat at concentrations that are approximately 30 % of maternal plasma levels [20]. A study investigating placental transfer of THC in Rhesus monkey’s found that THC was detectable in fetal plasma 15 min after infusion of the mother. Furthermore, fetal plasma levels of THC equilibrated to maternal levels within 3 h [19]. In this same study, mothers infused with 0.3 mg/kg of THC generated a peak THC concentration of 1419 ng/ml [19]. In contrast, a 70 kg woman consuming a cannabis chocolate bar containing 180 mg of THC would equate to THC consumption of 2.6 mg/kg.

The studies to be presented in this paper detail the effects of cannabinoids delivered at concentrations as low as 100 ng/ml. As discussed above, the potency of modern day cannabis product allows for maternal and fetal concentration of cannabinoids to easily exceed this threshold.

### Pre-implantation

**Fig. 2:**
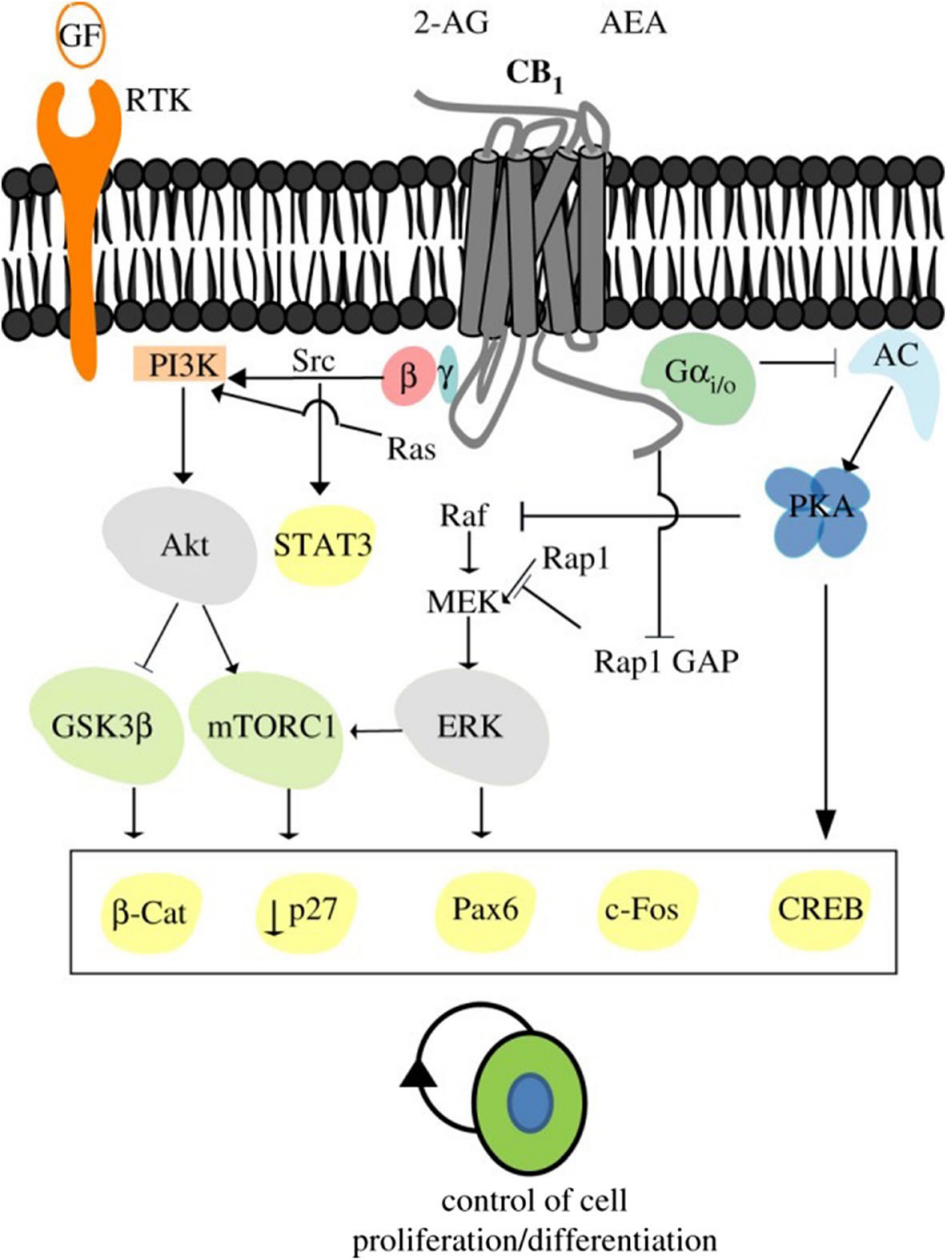
CB_1_ cannabinoid receptor signaling and regulation of neural stem/progenitor cell proliferation. CB_1_ receptors are coupled to G_i_ proteins, thereby mediating the inhibition of adenylyl cyclase (AC) and protein kinase A (PKA). CB_1_ receptor coupling to G_i_ signalling is also associated with activation of the extracellular signal-regulated kinase (ERK) pathway via different mechanisms. Direct activation of the PI3K/Akt and ERK pathways by CB_1_ receptors may converge, thus synergizing with their activation by other receptors such as growth factor receptors with tyrosine kinase activity (RTK). CB_1_ receptor-induced activation of RTKs can occur by promoting the processing of membrane-bound growth factor inactive precursors to yield active growth factors, or by activating intracellular Src family protein kinases. In some circumstances, CB_1_ activity can antagonize RTK-mediated ERK signaling. Activation of the CB_1_ receptor ultimately controls different transcriptional regulators, including CREB, STAT-3, PAX-6 and β-catenin. The CB_1_ receptor may also regulate mammalian target of rapamycin complex 1 (mTORC1) in NPs as it occurs in differentiated neurons. Permission to use figure was obtained from [44] and Creative Commons (https://creativecommons.org/licenses/by/4.0/)

THC is a component of the cannabinoid family, working mainly through the G protein-coupled receptors cannabinoid receptor type 1 (CB1) and type 2 (CB2) [21]. The activation of these receptors occurs via exogenous cannabinoids (i.e. THC) as well as endogenous cannabinoids (i.e. anandamide). Therefore, manipulation of endogenous cannabinoids can be used as a model for studying the effects of increased exogenous cannabinoid exposure. Both CB1 and CB2 are found in the female reproductive system and work in an inhibitory fashion by decreasing the activity of adenylyl cyclase and protein kinase A (Fig. 2). In terms of the effects of cannabinoid signaling on pre-implantation events, CB1 is of greater importance, as it is found in the oviduct, uterus, and from the late two-cell stage through the blastocyst stage of the embryo [1, 21, 22].

While translation of cannabinoid signaling studies in mice to humans have not yet been performed, evidence suggests that cannabinoid signaling plays an important role in the development of the mouse embryo. For example, in mouse models of *CB1-/-*, *CB2-/-*, and *CB1-/- x CB2-/-* double mutants, where there is no cannabinoid signaling, mutant embryos were recovered from the oviduct 3 and 4 days post fertilization showing asynchronous development relative to wild type [23]. Conversely, increased cannabinoid signaling is associated with developmental arrest of two-cell embryos in vitro in a dose-dependent manner [21]. Increased cannabinoid signaling via CB1 agonists has also been shown to lead to decreased blastocyst viability and a reduced number of trophectoderm cells, a decreased rate of zona-hatching, and inhibition of implantation [24]. Strong evidence supports the belief that these changes are associated with the CB1 mediated modulation of calcium channel activity, inhibition of adenylyl cyclase, activation of phospholipase C, and stimulation of mitogen-activated protein kinases (MAPKs) which include extracellular signal-regulated kinases (ERK), c-Jun N-terminal kinases (JNK), and p38 [1, 25–28]. In sum, deviations from homeostatic cannabinoid signaling highlight the importance of tightly regulated cannabinoid signaling for proper pre-implantation development.

In addition to the changes associated with increased cannabinoid signaling, there are changes in gene expression. In order to assess these changes, mice without fatty acid amide hydrolase (FAAH) were used. FAAH is involved with the degradation of anandamide, an endogenous cannabinoid, and therefore acts as a model for increased cannabinoid signaling. FAAH is also involved in regulating the magnitude and duration of anandamide signaling [29]. The *FAAH-/-* mice showed differential expression in genes related to cell migration most significantly, suggesting that cell migration in mice is defective with increased cannabinoid signaling [29, 30]. These findings support the changes associated with abnormalities that occur through the cell signaling pathways and CB1.

**Fig. 3:**
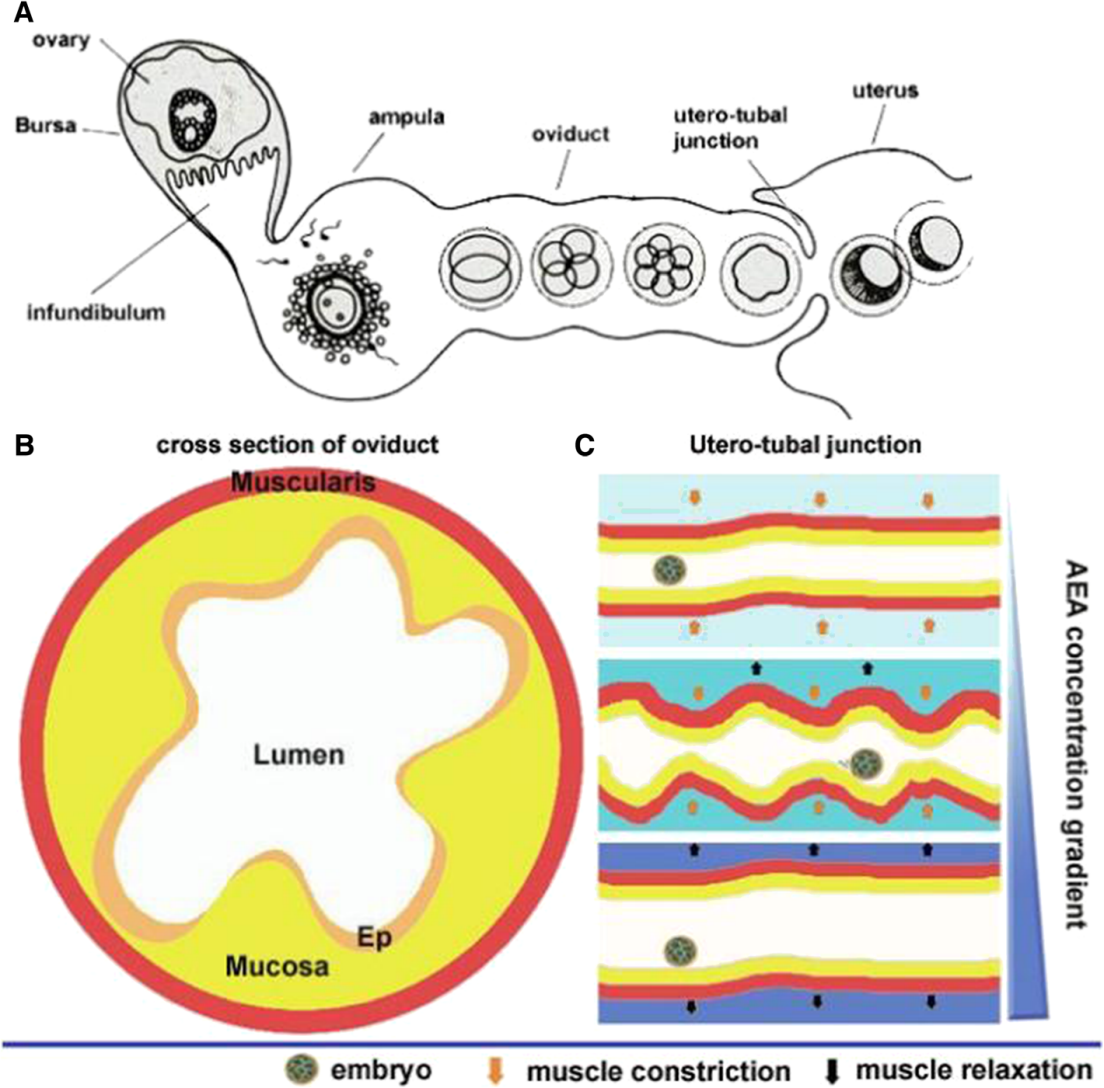
**a** A cartoon of oviductal transport of preimplantation embryos in mice. Ovulated eggs are fertilized in the ampulla of the oviduct. Fertilized eggs through successive cell divisions form morulae. Morulae pass through the utero-tubal junction to enter into the uterine lumen. **b** A schematic diagram of a cross-section of the oviduct. Ep, epithelium. **c** A proposed scheme of contraction–relaxation waves of oviductal muscularis at the utero-tubal region influenced by varying concentrations of AEA. In the absence of AEA, increased release of norepinephrine (NE) produces muscular contractions, resulting in narrowing of the lumen. At higher concentrations of AEA, endocannabinoid signaling through CB1 reduces the release of NE, thus relaxing the muscularis, widening the lumen. Thus, appropriate endocannabinoid signaling creates waves of contraction–relaxation, moving forward embryos from the oviduct into the uterine lumens [27]. With permission from Elsevier Inc

Along with proper embryological development, implantation is also dependent on timely transport of embryos from the oviduct to the uterus, as dysfunctional transport can increase the risk of ectopic pregnancy in women [31]. Figure 3a illustrates fertilization occurring in the ampulla followed by the subsequent divisions of the embryo in the oviduct as it travels towards the uterus. It is important to note that as the concentration of cannabinoids increases, there is an increase in muscle relaxation in the uterine lumen, as shown in Fig. 3c. However, as the cannabinoid concentration increases, there is also an associated loss of constriction that is essential for proper movement of the ovulated egg to the uterus for implantation [27, 31]. This observation is speculated to be facilitated by the presence of β-2 adrenergic and α-1 adrenergic receptors that cause sphincter relaxation and contraction, respectively [31]. CB1 expression in the muscularis of the oviduct is associated with the α-1 and β-2 receptors, leading to the speculation that cannabinoid signaling is functionally coupled to signaling through the adrenergic receptors, therefore facilitating oviductal motility [1, 31]. These notions are further supported as *FAAH-/-* mice, as well as wild-type mice with elevated exogenous cannabinoid ligands, observed impaired oviductal-uterine transport, which is defined as greater oviduct embryo retention [27, 31]. Consistent with current mouse data, venous blood obtained from women with ectopic pregnancies has been found to have significantly elevated cannabinoid levels when compared to normal pregnant controls, indicating that the link between altered cannabinoid signaling and impaired transport of the embryo most likely exists in humans [32]. This study found that mean anandamide levels (+/- SD) in the ectopic pregnancy group were 0.78 +/−0.04 nM, and healthy controls had anandamide concentrations of 0.63 +/−0.04 nM [32]. In comparison, smoking two marijuana cigarettes has been demonstrated to elevate plasma concentrations of THC by approximately 300 ng/mL [7].

While THC affects embryological development and transportation to the uterus, issues still arise from the process of implantation, illustrating the wide variety of ways in which increased cannabinoid signaling can alter implantation. In the mouse uterus specifically, the window of time for implantation is very limited such that it is receptive on day 4 post-fertilization, while it is considered non-receptive on day 5 [2, 24]. Implantation can be divided into the pre-receptive, receptive and refractory stages. The levels of endogenous cannabinoids are biphasic during this process as there are increased levels during the pre-receptive and refractory but low during the receptive [1, 27, 33]. Implantation fails when blastocysts are exposed to increased levels of cannabinoid signaling, ultimately blocking calcium channels and ERK activity [33]. Aside from the biphasic level of cannabinoid signaling during the implantation stage, this notion is supported as levels of cannabinoids at implantation sites are lower than inter-implantation sites with higher FAAH expression to promote the implanting process [2, 27, 33]. In sum, increased levels of CB1 activation through cannabinoid signaling during the receptive stage of implantation is associated with decreased uterine receptivity and reinforces the importance of tight regulation of this signaling pathway.

### Folic acid

Folic acid, also known as Vitamin B9, is essential for numerous bodily functions, including normal development and growth of the placenta and embryo [34]. It cannot be synthesized *de novo*, and thus must come from the diet or supplementation [34]. Folates are incredibly important within cells, as they function in one-carbon transfer reactions in growth and repair reactions, including the *de novo* biosynthesis of purine and pyrimidine precursors of nucleic acids, metabolism of amino acids and initiation of protein synthesis in mitochondria [35]. Folic acid is therefore vital to the process of DNA replication in dividing cells. Folic acid deficiency is associated with low birth weight, increased risk of spontaneous abortion, as well as neural tube defects such as spina bifida or anencephaly [35]. The process whereby folic acid travels from mother to embryo is critical to understanding the pathway through which THC and other cannabinoids may interfere with folic acid. Maternal to fetal folic acid uptake occurs transcellularly at polarized, epithelial-like syncytiotrophoblasts; this is the pathway that cannabinoids can disrupt [35].

Araújo et al. looked at the effects of cannabinoids on BeWo cells, which are a human choriocarcinoma cell line that can uptake folic acid, making them an appropriate placental synctiotrophoblast model [35]. This research showed that at physiological pH (7.5), the primary receptors involved in folic acid apical uptake are folate receptor α (FRα) and reduced folate transporter 1 (RFC1) [35]. FRα is a glycosylphosphatidylinositol-anchored protein, and is found on the apical side of the syncytiotrophoblasts [35]. FRα exhibits high affinity binding with folic acid, and is capable of internalizing it through receptor-mediated endocytosis [35]. On the other hand, RFC1 is a facilitative carrier that is driven by the transmembrane H+ gradient. Overall, RFC1 has a much greater affinity for reduced folates over non-reduced folates [34]. The proton-coupled folate transporter (PCFT) is another transporter that uses a high-affinity proton-coupled mechanism to translocate folates across the cellular membrane, acting optimally at acidic pH [35].

**Fig. 4:**
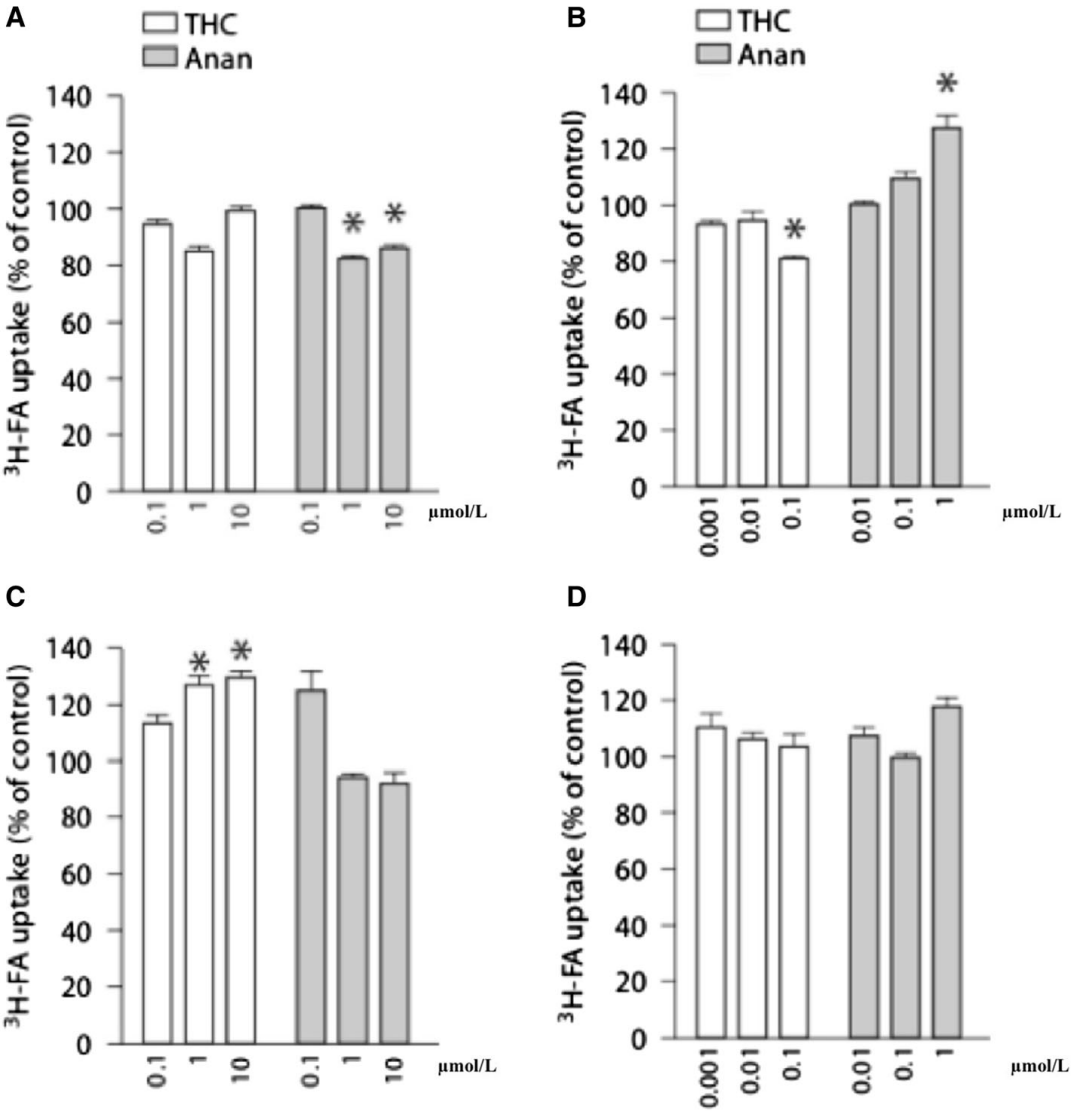
Acute effect of various cannabinoids upon the apical uptake of 3H-FA by BeWo cells (**a**, **b**). The cell monolayers were incubated at 37 °C for 6 min with 3H-FA (50 nmol/l) at pH 7.5 (**a**) or pH 6.5 (**b**) in the absence or presence of the compounds. The tested compounds were THC (*n* = 9) and anandamide (Anan; *n* = 9). 3H-FA uptake in control cells amounted to 0.73 ± 0.08 pmol/mg prot. (*n* = 18) (**a**) and 0.81 ± 0.07 pmol/mg prot. (*n* = 18) (**b**). The results are shown as arithmetic means ± SEM. *p < 0.05 compared to control condition. Chronic (48 h) effect of various cannabinoids upon the apical uptake of 3H-FA by BeWo cells (**c**, **d**). The cell monolayers were incubated at 37 °C for 6 min with 3H-FA (50 nmol/l) at pH 7.5 (A) or pH 6.5 (**b**) in the absence or presence of the compounds. The tested compounds were THC (*n* = 9) and anandamide (Anan; *n* = 9). 3H-FA uptake in control cells amounted to 0.44 ± 0.03 pmol/mg prot. (*n* = 15) (**a**) and 0.62 ± 0.09 pmol/mg prot. (*n* = 12) (**b**). The results are shown as arithmetic means ± SEM. *p < 0.05 compared to control condition. [Adapted from 35]. With Permission from Karger Inc. via RightsLink

**Fig. 5:**
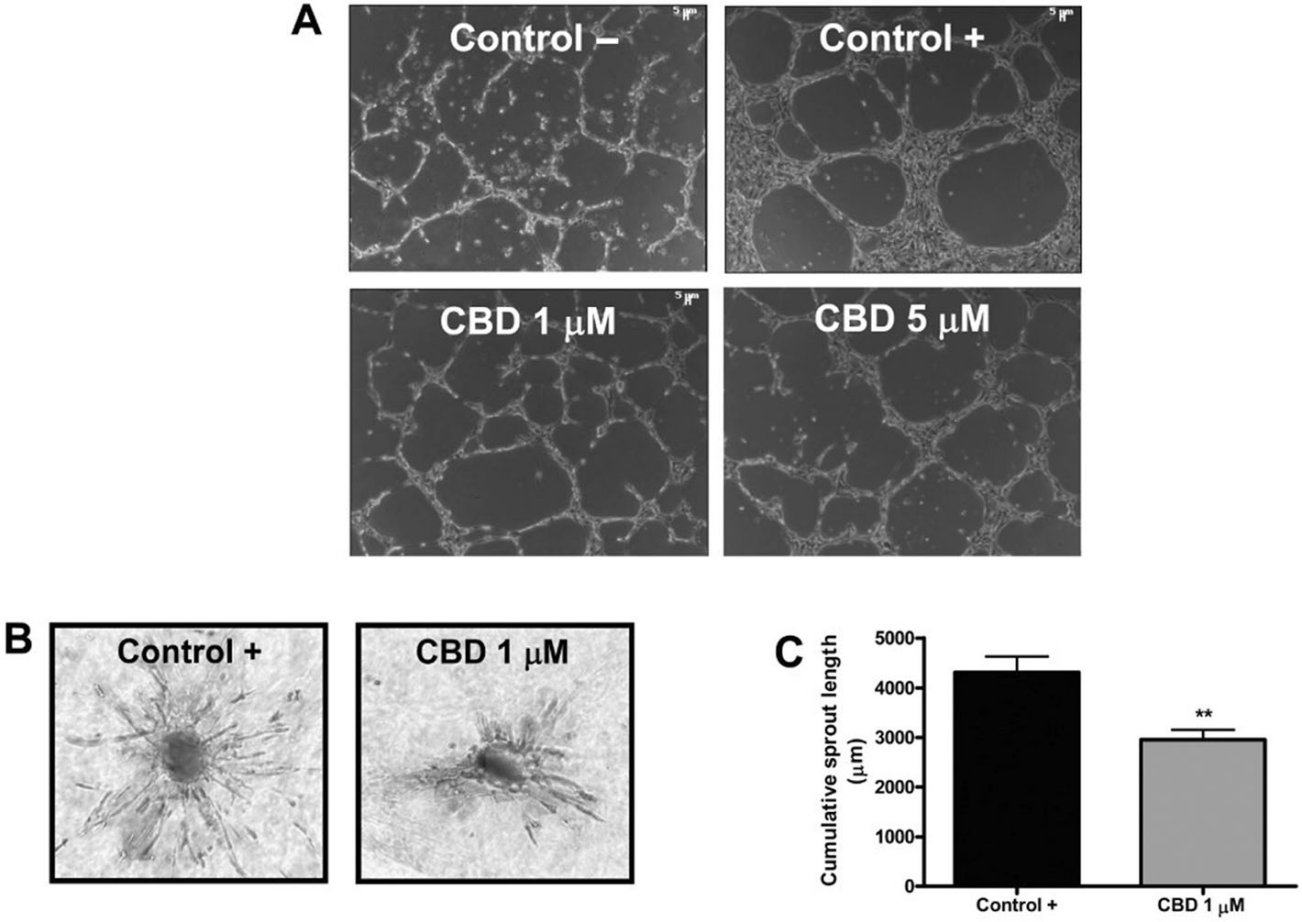
Cannabidiol inhibits in vitro endothelial morphogenesis and angiogenesis. **a** HUVECs were incubated on a Matrigel substrate in the presence of M199 alone (Control –) or of M199 supplemented with 2 % FBS (Control +), in the absence or presence of different concentrations of CBD for 6 h at 37 °C. CBD interfered with HUVEC organization in capillary-like networks. **b** HUVEC spheroids, were embedded in collagen gel supplemented with VEGF (30 ng · mL-1) in the absence (Control) or in the presence of CBD (1 mM). Representative photos of each experimental group are shown. **c** Quantification of the sprouting. The results are expressed as the mean ± SEM of the cumulative sprout length of the capillary-like structures emerging from 24 to 26 individual spheroids per experimental group. **p < 0.01 compared to spheroids from control HUVECs, Dunnett’s *t*-test [Adapted from 41]. With permission from Wiley and Sons Inc. via RightsLink

Araújo et al’s results ultimately demonstrated that acute cannabinoid consumption resulted in no effect on the apical uptake of folic acid, while the chronic (48 h) effect of THC manifested itself in a significant decrease in folic acid uptake [35]. Figure 5a and b shows the effect of acute cannabinoid consumption on folic acid uptake. These cells were incubated at 37 °C for 6 min with folic acid in the absence or presence of THC or anandamide. The results show that at physiological pH of 7.5, anandamide caused a 15 ± 4 % decrease in uptake, while THC had no effect on uptake [35]. Figure 4 also shows the effects of chronic cannabinoid consumption on folic acid uptake. At physiological pH of 7.5, 100 nmol/L of THC is shown to cause a significant folic acid uptake decrease of 20 ± 4 %. These data ultimately demonstrate that in BeWo cell lines, chronic consumption of THC results in a markedly decreased folic acid uptake. This suggests that marijuana use during pregnancy could result in folic acid deficiencies and possibly developmental defects.

### Cellular growth factors

Cannabinoid exposure poses an acute risk to the developing human embryo through its ability to interfere with crucial modulators of cellular growth and angiogenesis such as Vascular Endothelial Growth Factor (VEGF), by its ability to restrict movement of Human Umbilical Vein Endothelial Cells (HUVEC), and by its ability to induce apoptosis across multiple cell lineages.

VEGF is considered the most important proangiogenic molecule due to its expression in a wide variety of human tissues, and because of potency and ability to regulate perhaps every step of the angiogenic cascade [36–39]. Numerous investigations have illustrated that cannabinoid exposure reduces VEGF expression across multiple cell lineages in both a dose-dependent and time-dependent manner [40, 41]. For example, injection of the CB2 agonist JWH-133 (JWH), a non-psychoactive cannabinoid, in mice bearing subcutaneous gliomas resulted in a reduction of the expression of 10 genes that are either directly or indirectly related to the VEGF pathway [36]. The same experiment also showed that cannabinoid injection resulted in a 50 % reduction in expression of VEGF-A and an 80 % reduction in expression of VEGF-B [36]. Additionally, Solinas et al. demonstrate that exposing HUVEC cells to Cannabidiol (CBD), a peripherally active cannabinoid, significantly inhibited the VEGF induced outgrowth of capillary-like structures (Fig. 5.) [41]. It is proposed that the mechanism of this action is via a ceramide-dependent cannabinoid-induced inhibition of the VEGF receptor 2 (VEGFR-2) action [36].

Cannabinoid administration to cell lines also disrupts cellular functions such as DNA replication and cellular motility in vivo *and* in vitro. Treatment of androgen-sensitive human prostate adenocarcinoma cells (LNCaP) with WIN-55,212-2 (WIN), the cannabinoid and CB1 agonist, results in a significant decrease in the expression of Proliferating Cell Nuclear Antigen (PCNA), an essential processivity factor for DNA replication [40]. With respect to cellular motility, it is found that administration of cannabinoids to HUVEC in vitro results in a statistically significant decrease in cellular migration, although no appreciable apoptosis was observed [41]. Given that cellular migration and replication are crucial processes to a developing embryo and proliferating fetal tissues, cannabinoid exposures during the fetal development process may result in adverse outcomes.

In vivo and in vitro exposure to cannabinoids induces apoptosis across numerous cell lineages. Blazquez et al. find that HUVEC death occurs when cultured cells are treated with WIN [42]. Similarly to WIN’s effect in umbilical stem cells, in vivo cannabinoid-induced apoptosis has been replicated in both the PDV.C57 epithelial tumor cell line and in treatment of LNCaP cells [40, 43]. The cellular environment of a growing tumor closely models that of a developing embryo– an environment of rapidly proliferating and differentiating tissues seeking vascular access for growth. While apoptosis of tumor cells is the desired effect of cancer treatment, embryological growth requires viable cells and vascular proliferation. In sum, the ability of cannabinoids to induce apoptosis in both tumor cells lines and HUVEC lines may translate to an analogous and undesired apoptotic effect in a developing embryo.

### MAPK/ERK and neural development

The regulation of neuronal cell differentiation and growth occurs through various signaling pathways in the embryo and is critical to proper fetal brain development. Type I cannabinoid receptors are expressed abundantly in the fetal brain, suggesting they play a vital role in the development of the embryologic neural system [44]. Neural stem cells (NSCs), the precursor cells in development, can differentiate into either neurons or glial cells, such as astrocytes or oligodendrocytes, by the process of neurogenesis during embryo development [45]. Research suggests that CB1 receptor activity by cannabinoids has an important regulatory role in determining the fate of neural cells, through processes such as cell cycle progression and proliferation, specification, axonal migration and morphogenesis [44]. While current research suggests varying, and at times, contradictory effects of cannabinoids on neural development and highlights the need for more advanced studies, the importance of such research is to show that exogenous cannabinoids can disrupt the underlying homeostasis required for proper development to occur.

The role of the CB1 receptor in neuronal progenitor cell differentiation was investigated using NSCs obtained from the cerebral cortexes of mouse embryos and treatment with endocannabinoids, including anandamide and arachidonyl-2’-chloroethylamide (ACEA) [45]. The results of this study indicated that when exposed to anandamide or ACEA, NSC differentiation into neurons is promoted, in comparison to the control. This finding is indicated by increased cell expression of β-III tubulin, a neuronal marker, over GFAP, an astrocyte marker, as well as decreased expression of Nestin, a neuronal precursor marker indicating the undifferentiated state. In addition, ACEA-treated NSCs showed a higher degree of maturation and complexity after differentiation, illustrated by increased neuronal length, number of neurites, and branching [45]. Studying the expression of genes involved in the differentiation pathways suggested that activation of the CB1 receptor by endocannabinoids increased expression of genes promoting neuronal cell fate and decreased those genes that promote further NSC proliferation in vivo [45]. Therefore, the role of endocannabinoids in altering rate, commitment and morphology of NSC differentiation could be detrimental to this tightly regulated and sensitive process of neural development.

Interestingly, similar evidence regarding neuronal differentiation has also been observed with the chronic treatment of adult rat hippocampal NSCs with HU210, a synthetic cannabinoid, and anandamide [46]. A significant increase in newborn neurons was observed in the hippocampal dentate gyrus, a location that is capable of producing a large increase in neurons from its existing NSC population in adults [47]. While this observation may prove useful in the future treatment of neurological disorders, such unregulated neuron proliferation deviates from embryological homeostasis.

The Mitogen-activated protein kinase (MAPK) signaling pathway has been studied in the endocannabinoid dysregulation of NSC differentiation. The MAPK family is highly conserved in the control of numerous cellular processes, such as proliferation, differentiation and the stress response [28]. Three specific subfamilies of MAPK have been studied in relation to cannabinoids- p38-MAPK, c-Jun N-terminal kinases (JNK) and extracellular signal-regulated kinase (ERK), with the most extensive research focusing on ERK.

The p38-MAPK and JNK subfamilies are involved in the cellular stress response, which is important in the developing embryo. Rat and mouse hippocampal slices exposed to both endogenous (anandamide) and exogenous (THC) cannabinoids exhibited an increase in p38-MAPK phosphorylation, but no effect on JNK [28]. The activation of p38-MAPK was discovered to involve CB1 receptors, but the mechanism is still unknown; this activation of p38-MAPK was observed in adult hippocampal slices. Since both exogenous and endogenous cannabinoids can cross the blood-placental barrier, this raises the concern that this may be observed in the embryo. This is significant because p38-MAPK activity acts as a switch for the commitment of embryonic stem (ES) cells to neurogenesis (p38 not expressed) and cardiomyogenesis (p38 expressed) early in embryo development, and also acts differentially throughout the developmental stages [48]. Thus disregulation of the p38-MAPK signaling pathways can be detrimental to ES cell commitment and differentiation.

**Fig. 6:**
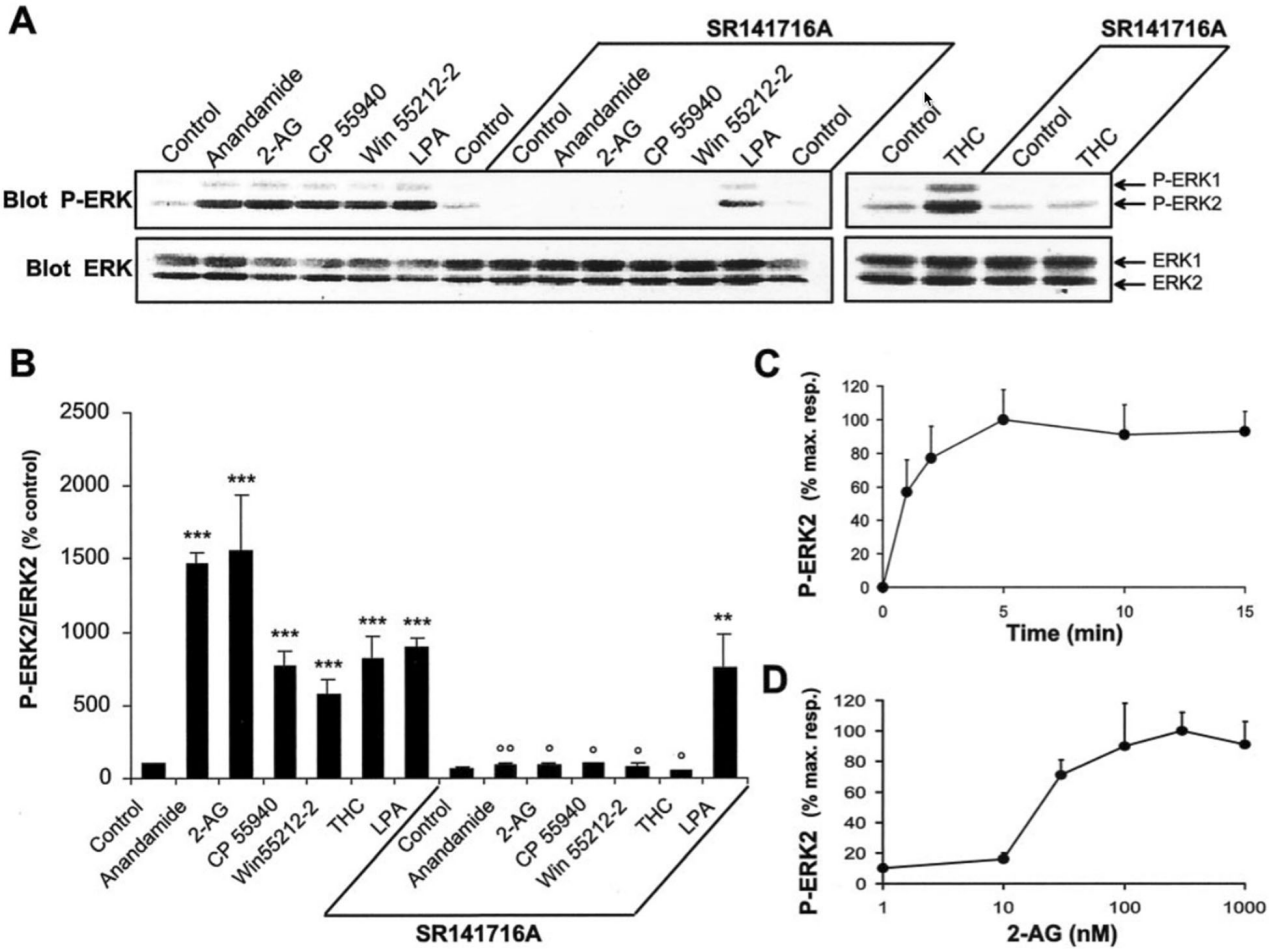
Cannabinoid agonists stimulate ERK phosphorylation in rat hippocampal slices. **a** Rat hippocampal slices were incubated at 35 °C, as described in Materials and Methods, for 50 min before the addition of vehicle (*Control*), 1 μM anandamide, 1 μM 2-AG, 1 μM CP 55940, 100 μM WIN 55212–2, 0.2 μM LPA, or 0.1 μM Δ9-THC for 5 min, in the absence or in the presence of 100 μM SR 141716A applied 30 min before. Slices were homogenized in SDS; 60 μg of protein per sample were subjected to immunoblot analysis using antibodies specific for the dually phosphorylated (active) forms of ERK1 and ERK2 (*Blot P-ERK*). After stripping, the membranes were reprobed with anti-ERK (*Blot ERK*) antibodies. **b** For quantification the optical densities of P-ERK2-immunoreactive bands were measured, normalized to the optical densities of total ERK2 in the same samples, and expressed as percentages of controls. Data correspond to means ± SEM. Statistical analysis was done with ANOVA (*F*_(13,24)_ = 22.8; *p* < 0.0001) followed by *t* test (treated vs control: ***p < 0.001, **p < 0.01; treated in the presence of SR141716A vs in its absence: °° *p* < 0.01, ° *p* < 0.05). **c**, **d** Quantification of the effects of 2-AG on ERK2 active form: time course (drug concentration 1 μM) (**c**); concentration–response curve (treatment for 5 min) (**d**). Immunoreactivity was quantified by scanning densitometry using NIH image 1.62 software. Values are means ± SEM of four to eight independent experiments and are expressed as percentages of the maximal increase above unstimulated control values [49]. With open permission from the Journal of Neuroscience

Experiments in vitro using rat and mice hippocampal slices and in vivo in mice were performed to investigate the exposure of cannabinoid agonists on ERK regulation [49]. The hippocampus is vital in learning and memory, making it possible that *in utero* disruptions to its formation via cannabinoid exposure could manifest post-natally. The results showed a significant increase in ERK1 and ERK2 activation by phosphorylation in the hippocampus when exposed to cannabinoids, including both anandamide and THC (Fig. 6). When the CB1 receptor gene was knocked out, ERK activation was not observed, helping to confirm that agonists acted on the receptor [49].

**Fig. 7:**
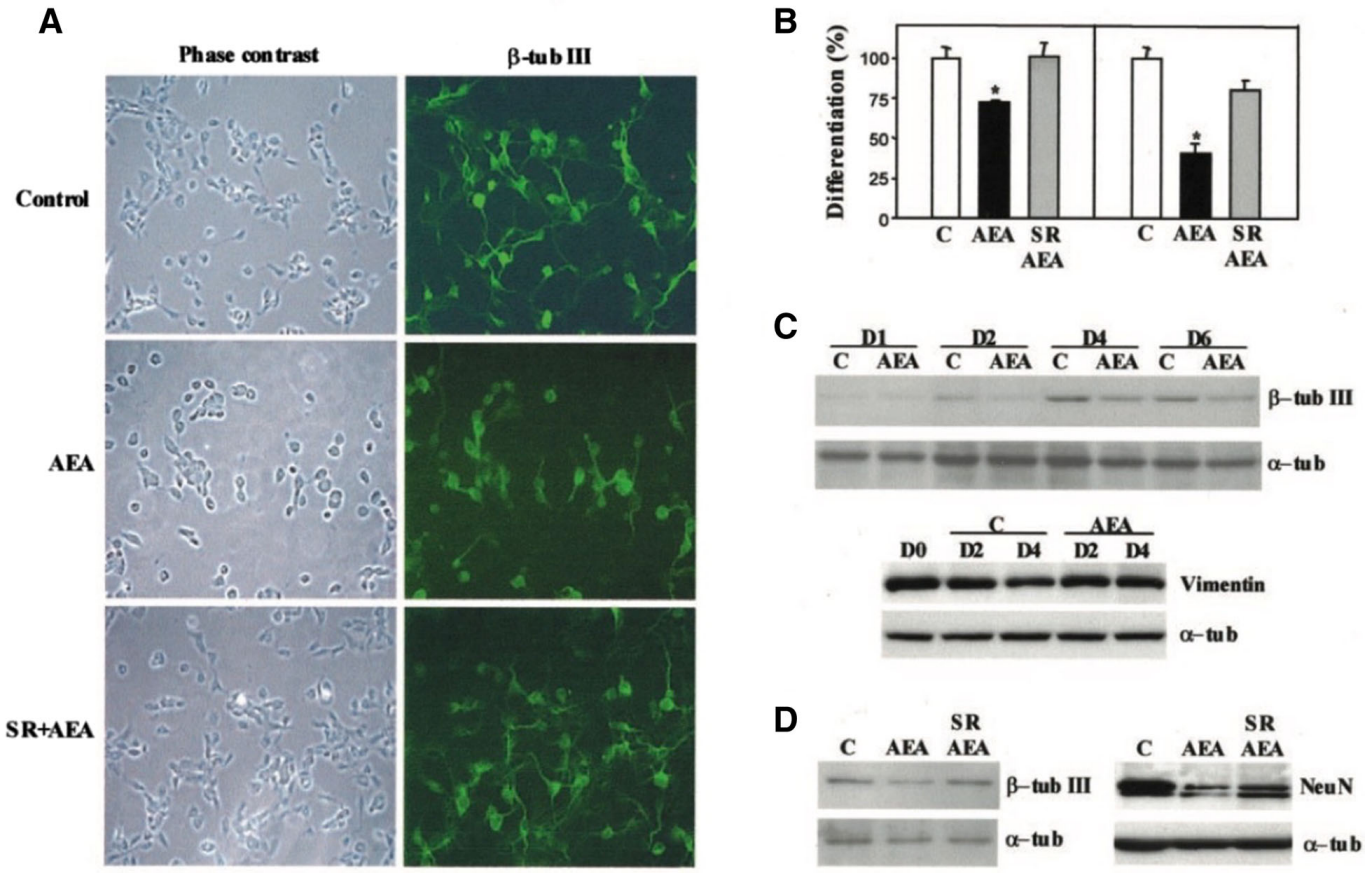
Anandamide inhibits neuronal differentiation via CB_1_. E17 cortical neuron progenitor differentiation was performed in the presence of the indicated agents. **a** Immunofluorescence with anti-β-tubulin III antibody after 24 h of 5 μM AEA incubation in the absence or presence of 2 μM SR141716. **b** Percentage of neurons bearing neurites longer than twice (*left panel*) or five times (*right panel*) the cell body after quantification of immunofluorescence photographies. **c** Western blot of β-tubulin III and vimentin expression during the indicated days of cortical neuron development in the absence or presence of AEA. Loading controls were carried out with an anti-α-tubulin antibody. **d** Western blot of β-tubulin III (*left panel*) and Neu N (*right panel*) in cortical neuron extracts from cultures in the absence or presence of AEA and prevention of the AEA effect by SR141716. Results correspond to four different experiments. Significantly different from controls *p < 0.01 [50]. With Permission from the Journal of Biochemistry

As extensive research on THC’s effects on the embryo is lacking, cannabinoids have also been reported to inhibit the ERK pathway within the hippocampus [50]. Using cortical neuron progenitors taken from 17-day-old rat embryos previously exposed to chronic administration of anandamide for 2 weeks, decreased neuronal differentiation was observed. The CB1 receptor activity of endocannabinoids was determined to inhibit ERK activation in the dentate gyrus of the rat hippocampus (Fig. 7) [50]. These results are seemingly contradictory to evidence that showed cannabinoids activated the ERK pathway in mice hippocampus both in vitro and in vivo [49]. However, this varying evidence may be due in part to different experimental conditions and cannabinoid exposure amounts. This highlights the sensitivity of the neural development processes to the effects of compounds appearing in concentrations varying from their homeostatic concentrations.

Similarly, the reported inhibition of NSC differentiation due to the reduction of the ERK pathway appears contradictory to evidence showing an increase in neural differentiation upon cannabinoid-induced reduction of the ERK pathway [45, 50]. These contradictory results may be in part explained by the differing roles of the ERK pathway in various parts of the neural system development, as the studies analyzed hippocampi and cerebral cortices respectively. This evidence significantly shows that neural development is tightly regulated and varies greatly in different locations, and therefore cannabinoid exposure disruption of the signaling processes critical to this development could be detrimental and must be further studied.

The MAPK/ERK pathway regulates the gene expression through the activation of transcription factors and thus indicates many ways development could be altered [51]. ERK pathway regulation has been implicated in neural processes, such as synaptic plasticity, learning and memory, as well as general processes, such as gene transcription, translation and cytoskeletal rearrangement [49, 52]. While it is still unclear what role exactly cannabinoids play in NSC differentiation and the ERK pathway in different parts of the neural system, any alteration from normal homeostatic pathway levels at such a critical time and location in development is significant nonetheless and should be a target of future research [49, 50]. Furthermore, MAPK signaling is involved in the broader RAS-MAPK pathway downstream of receptor tyrosine kinase signaling, which is involved in cell fate decisions of developmental processes such as neural patterning and limb development [52].

THC also induces brain-derived neurotrophic factor (BDNF) mRNA transcription through the ERK signaling pathway [51]. BDNF is a growth factor that behaves as an immediate-early gene involved in synaptic efficiency, neuronal survival and new neuron differentiation, as well as in long-term memory. While decreased BDNF is associated with certain pathologies, including depression, increased expression has been correlated to *in utero* valproic acid (VPA) exposure [53]. VPA is an antiepileptic and mood-stabilizing drug, and its stimulation of BDNF expression when introduced *in utero* is associated with increased risk of congenital malformations and impaired cognition, including autism and low IQ. By studying fetal mice brains exposed to VPA, it has been suggested that the embryonic brain, which normally contains low BDNF levels, is highly sensitive to both increases and decreases in the protein, and that disrupting this tight regulation can alter prenatal cortical development by altering NSC proliferation and differentiation [53]. Therefore, future research should look to assess whether the THC-induced increase in BDNF expression mimics the post-natal outcomes of the VPA-induced BDNF stimulation.

The MAPK/ERK pathway also plays a role in the formation of the blood-placental barrier [51]. Using mouse embryos and placentae, it was determined that normal MAPK/ERK signaling is required to form the syncytiotrophoblast that make up the blood-placental barrier. The barrier allows for separation of fetal and maternal circulation within the placenta and is vital for embryo development. Therefore, deviations from the normal signaling pathway represents another means by which cannabinoids could potentially alter development, although this has yet to be reported on in the literature.

In embryogenesis, ERK signaling between tissues may be important in the ultimate 3D organization. In mouse embryos, dephosphorylated forms of ERK1/2 were used to map out domains of signaling activation during embryogenesis [52]. Activated ERK domains were found at all stages of embryo development, with variations in pattern complexity and activation time. For example, while CNS domains showed more sustained ERK activation, many processes such as blood vessel formation, neural crest migration, somite remodeling and PNS development showed dynamic pulses [52]. This indicates that spatial and temporal patterns of ERK activation are highly regulated with regards to timing, location, intensity and duration to ensure proper shaping of the embryo, and thus disrupting this precise signaling may alter these processes.

## Conclusions

Under normal physiologic conditions, cannabinoid signaling exhibits a wide-range of downstream effects embryologically, as is illustrated by Fig. 1. This breadth of action ultimately manifests in the symptoms associated with cannabis use during pregnancy, including miscarriage, congenital malformations, and learning disabilities [1]. Cannabinoids have received renewed attention in the field of cancer treatment due to their pharmacologic activities in vivo as cell growth inhibitors, restrictors of cellular motility, and their ability to induce apoptosis across multiple cell lineages [40, 41]. However, by the same mechanisms that cannabinoids show promise in the field of cancer treatment, they prove equally dangerous to the viability and health of a developing embryo. A summary of these mechanisms is provided in Table 1.

The effects of cannabinoids on pre-implantation and embryologic development have the potential to elicit harmful outcomes post-natally. While the method of consumption can affect the severity of the embryologic effects, it is important to note that this paper is an overview of the effects solely of elevated cannabinoid signaling. A common means of cannabis consumption is smoking, which can add a number of toxins and thereby amplify harmful effects to the embryo. Given the trend of marijuana decriminalization and legalization across the United States, further epidemiological research should focus on the association between maternal cannabinoid use and observation post-natal outcome.
